# Racial and ethnic disparities in the control of cardiovascular disease risk factors in Southwest American veterans with type 2 diabetes: the Diabetes Outcomes in Veterans Study

**DOI:** 10.1186/1472-6963-6-58

**Published:** 2006-05-23

**Authors:** Christopher S Wendel, Jayendra H Shah, William C Duckworth, Richard M Hoffman, M Jane Mohler, Glen H Murata

**Affiliations:** 1 Southern Arizona VA Health Care System, Tucson, AZ, 85723, USA; 2 University of Arizona College of Medicine, Tucson, AZ, 85724, USA; 3 Carl T. Hayden VA Medical Center, Phoenix, AZ, 85012, USA; 4 New Mexico VA Health Care System, Albuquerque, NM, 87108, USA; 5 University of New Mexico School of Medicine, Albuquerque, NM, 87131, USA

## Abstract

**Background:**

Racial/ethnic disparities in cardiovascular disease complications have been observed in diabetic patients. We examined the association between race/ethnicity and cardiovascular disease risk factor control in a large cohort of insulin-treated veterans with type 2 diabetes.

**Methods:**

We conducted a cross-sectional observational study at 3 Veterans Affairs Medical Centers in the American Southwest. Using electronic pharmacy databases, we randomly selected 338 veterans with insulin-treated type 2 diabetes. We collected medical record and patient survey data on diabetes control and management, cardiovascular disease risk factors, comorbidity, demographics, socioeconomic factors, psychological status, and health behaviors. We used analysis of variance and multivariate linear regression to determine the effect of race/ethnicity on glycemic control, insulin treatment intensity, lipid levels, and blood pressure control.

**Results:**

The study cohort was comprised of 72 (21.3%) Hispanic subjects (H), 35 (10.4%) African Americans (AA), and 226 (67%) non-Hispanic whites (NHW). The mean (SD) hemoglobin A1c differed significantly by race/ethnicity: NHW 7.86 (1.4)%, H 8.16 (1.6)%, AA 8.84 (2.9)%, p = 0.05. The multivariate-adjusted A1c was significantly higher for AA (+0.93%, p = 0.002) compared to NHW. Insulin doses (unit/day) also differed significantly: NHW 70.6 (48.8), H 58.4 (32.6), and AA 53.1 (36.2), p < 0.01. Multivariate-adjusted insulin doses were significantly lower for AA (-17.8 units/day, p = 0.01) and H (-10.5 units/day, p = 0.04) compared to NHW. Decrements in insulin doses were even greater among minority patients with poorly controlled diabetes (A1c ≥ 8%). The disparities in glycemic control and insulin treatment intensity could not be explained by differences in age, body mass index, oral hypoglycemic medications, socioeconomic barriers, attitudes about diabetes care, diabetes knowledge, depression, cognitive dysfunction, or social support. We found no significant racial/ethnic differences in lipid or blood pressure control.

**Conclusion:**

In our cohort, insulin-treated minority veterans, particularly AA, had poorer glycemic control and received lower doses of insulin than NHW. However, we found no differences for control of other cardiovascular disease risk factors. The diabetes treatment disparity could be due to provider behaviors and/or patient behaviors or preferences. Further research with larger sample sizes and more geographically diverse populations are needed to confirm our findings.

## Background

Type 2 diabetes causes a substantial burden of suffering for minorities. Compared to non-Hispanic whites, all minorities except Alaskan natives have a 2- to 6-fold increased risk of acquiring the disease [1-3], and the prevalence is rising in some groups, including African Americans, Hispanic Americans, and American Indians [4]. Many population studies have shown that minorities do not achieve the same level of glycemic control as non-Hispanic whites [3,5-8]. Cultural and socioeconomic differences may create barriers to health by making it difficult to adhere to customary self-care recommendations for diet, weight loss, exercise, or blood glucose monitoring [1,2,9-11]. Finally, minorities have been shown to have an increased risk of developing micro- or macrovascular disease complications [1,12,13]. An excess risk has been described for nephropathy [3,14-16], retinopathy [17-19], amputations and foot problems [1,20], coronary artery disease [3], and stroke [3,21] compared to non-Hispanic whites. This health care burden makes it imperative to develop appropriate interventions for minority patients.

Evaluating racial and ethnic variations in the intermediate outcomes of diabetes care may be the best way to assess diabetes management. A comprehensive evaluation is essential because socio-cultural barriers may differentially influence various clinical outcomes. Evaluations should encompass weight control, diet, exercise, glycemic control, smoking status, lipid management, and blood pressure control. The American Southwest is an appropriate region for evaluating racial and ethnic differences in diabetes care because of the high prevalence of disease and the large minority populations. The purpose of this study was to examine the association between race/ethnicity and control of cardiovascular disease risk factors, adjusted for socioeconomic, clinical, and behavioral factors, in a large cohort of insulin-treated veterans with type 2 diabetes.

## Methods

The Diabetes Outcomes in Veterans Study (DOVES) was a prospective, observational study of insulin-treated veterans with type 2 diabetes mellitus, designed to examine the association between clinical, demographic, lifestyle, socioeconomic, and psychological variables and the clinical outcomes of glycemic control and disease management. DOVES was conducted under the auspices of the Southwestern Group for Outcomes Research in Diabetes (SWORD), a consortium of the largest VA facilities in Veterans Integrated Service Network 18. The protocol for this study was described in detail elsewhere [22]. The institutional review board of each study site approved the protocol. Briefly, computerized pharmacy records were used to identify insulin-treated patients receiving care at the New Mexico VA Health Care System (Albuquerque, NM), the Carl T. Hayden VA Medical Center (Phoenix, AZ), and the Southern Arizona VA Health Care System (Tucson, AZ). Patients were eligible for this study if they had type 2 diabetes diagnosed after age 35; took at least daily one injection of a long-acting insulin preparation, did not self-titrate their insulin doses; and their diabetes medication regimen had been relatively unchanged in the preceding 2 months (the dose of all oral hypoglycemic medications remained unchanged, no oral hypoglycemic medications were added to the treatment regimen, and the total daily insulin dose was changed by no more than 10 units or 15%, whichever was less), thus ensuring that their A1c had equilibrated at the current insulin dose.

We excluded patients with less than a one-year expected survival; alcoholism or substance abuse listed as an active problem in the electronic medical record; a history of diabetic ketoacidosis or type I diabetes; or co-morbidities affecting glucose homeostasis: diabetes resulting from pancreatitis or pancreatic resection; cirrhosis, chronic active hepatitis, hemochromatosis, Wilson's disease or other liver disease; endocrinopathies such as pituitary adenoma, Cushing's or Addison's disease; hereditary or acquired forms of insulin resistance; glucocorticoid treatment; immunosuppression or treatment with immunosuppressant drugs; or chronic infectious diseases (e.g. osteomyelitis or refractory skin ulcers). We also excluded homeless patients because they would have difficulty with the intensive self-monitoring of blood glucose required for the DOVES prospective study.

All measures in this report were collected at the two baseline visits, scheduled two weeks apart, with the exception of insulin doses measured at a 26-week follow-up visit. At the entry visit, research coordinators explained the project, answered questions, and obtained informed consent. All subjects underwent an evaluation of their psychological status, socio-cultural barriers to diabetes management, disabilities, dietary habits, exercise patterns, and micro- and macrovascular disease risk factors. Race/ethnicity was determined from a structured category question that asked the patient to describe his or her background. The listed responses included non-Hispanic white, Hispanic, Native American, African-American, and Asian. Subjects also had the option to check an "other" category and write in a response. Subjects then answered questions on their family obligations, living arrangements, means of transportation, occupation, and financial status. Subjects rated their physical ability to do the following activities: work, yard work, household projects, shopping, exercise, cooking or light housekeeping, and personal care. Research coordinators conducted structured interviews with study subjects to collect data about medical treatment, including insulin dose, number of injections, types of preparations, and the dose, type, and frequency of use of oral hypoglycemic medications. Research coordinators then ascertained the number of units of each insulin type at each daily dosing time and summed them to determine total insulin units per day.

Psychosocial testing was performed in private sessions during the baseline visit and the second visit two weeks later. A research coordinator was present to provide instructions, answer questions, clarify items, or in some cases, read the questions. Psychological instruments were administered in random order and included the University of Michigan Diabetes Knowledge Test [23], the Mini-Mental State Examination (MMSE) [24], the Geriatric Depression Scale [25], the Diabetes Family Behavior Check List [26], and the Diabetes Care Profile [27]. We used the Compendium of Physical Activities to rate physical activities [28] and the Fred Hutchinson Cancer Research Center Food Frequency Analysis to assess dietary habits [29]. Baseline physiologic measurements made upon entry to the study included hemoglobin A1c (A1c), blood pressure, height, weight, smoking status, and blood lipids.

### Statistical analyses

We analyzed group differences in continuous variables by the unpaired Student's t-test and one-way analysis of variance. For the latter procedure, homogeneity of variances was examined by Levene's test, and the Brown-Forsythe test was used in place of the standard ANOVA F-test if the variances were significantly different (performed on BMDP software). The Mann-Whitney U-test and the Kruskal-Wallis one-way analysis of variance by ranks were used for variables with highly skewed distributions. Group differences in nominal variables were tested by chi-square analysis. The relationship between continuous variables was examined by simple regression. We used stepwise multiple linear regression analyses to identify factors affecting the baseline A1c and daily insulin doses. Predictors associated with the dependent variable in univariate analysis (p < 0.10) were entered into multivariate model using a forward- and backward-stepping procedure with an α ≤ 0.05 to enter and an α > 0.10 to remove. Continuous variables were expressed as mean ± standard deviation (SD).

## Results

We identified over 10,000 insulin-treated patients at the three participating medical centers. We randomly selected approximately 3000 subjects from this list and invited 589 eligible subjects to participate. We enrolled 359 (61%) subjects, but subsequently excluded 21 with incomplete data, leaving a cohort of 338 for the analysis. Their mean age was 65.1 ± 9.7 years, 96% were men, and 59% were married. Based on self-description, the cohort was comprised of 226 (67%) non-Hispanic white, 72 (21%) Hispanic, and 35 (10%) African-American subjects. Over two-thirds of the subjects had at least one microvascular disease complication and an equivalent number had a macrovascular disease complication. Although average daily insulin doses were substantial (66 units), only one-third of the subjects were concurrently treated with an oral hypoglycemic medication. Most subjects had an elevated baseline A1c value suggesting poor glycemic control, including 99 (29%) with a value between 7.0% and 8.0%, and 148 (44%) with a value ≥ 8.0%. The median level of activity was 7 met-hours of activity per day (equivalent to 2.5 hours of light home activities plus 0.5 hours of moderate walking). Sixty-two percent had a BMI ≥ 30 and 22.2% were current smokers. However, average lipid and blood pressure measurements were close to the target values recommended by the American Diabetes Association (ADA) [30,31].

Clinical and socioeconomic characteristics stratified by race/ethnicity are presented in Table 1. African-American subjects had the highest A1c and were most likely to have poor glycemic control. Fifty-one percent of African Americans had an A1c ≥ 8% compared to 49% for Hispanics and 40% for non-Hispanic whites (p = 0.27). Mean ± SD A1c levels were 7.9 ± 1.4% for non-Hispanic whites, 8.2 ± 1.6% for Hispanics, and 8.8 ± 2.9% for African Americans (P = 0.05). We found significant differences in the daily units of insulin, with non-Hispanic whites receiving 70.6 ± 48.8 units compared to 58.4 ± 32.6 units for Hispanics and 53.1 ± 36.2 units for African Americans (p < 0.01). However, we found no differences between minorities and non-Hispanic whites in the daily number of insulin injections, the number of different insulin preparations used, or the use of oral hypoglycemic medications. Body mass index, amount of exercise, smoking status, lipid levels, and blood pressures were also similar between groups.

We also evaluated potential barriers to care. African-American subjects considered themselves less disabled for work (Table 1). Hispanic subjects had the most dependents, and reported the highest psychosocial barriers with respect to language preference, education, depression, diabetes knowledge, and performance on the MMSE (Table 2). African-Americans perceived the fewest problems with glycemic control, had the fewest negative attitudes about diabetes, and had the highest self-ratings for self-care abilities and dietary adherence. They also tended to have the strongest convictions about the importance of self-care and the fewest perceived barriers to exercise.

We found that higher depression scores (r = 0.11, p = 0.049), greater work hours per week (r = 0.17, p = 0.002), greater number of household dependents (r = 0.13, p = 0.023), being employed (p = 0.004), and age (r = -0.21, p = 0.0001) were significantly associated with a higher baseline A1c. After adjusting for these covariates with a multivariate linear regression analysis, baseline A1c was significantly higher for African-American subjects (+0.93%, p = 0.002), though not for Hispanics (+0.25%, p = 0.29), compared to non-Hispanic whites.

We stratified daily insulin dose by race and ethnicity and level of glycemic control (Table 3). We found that unadjusted daily insulin units increased monotonically with A1c in non-Hispanic whites, but not in Hispanics or African Americans. We found the most significant racial/ethnic differences in insulin doses among subjects with poorly controlled diabetes (A1c ≥ 8.0%), with non-Hispanic whites receiving approximately 22 daily units more than Hispanics and 26 daily units more than African Americans (p < 0.01). At follow-up, the treatment patterns remained essentially the same. Overall, 63% of each minority group and 70% of the non-Hispanic whites returned for the 26-week follow-up visit. For non-Hispanic whites, Hispanics, and African Americans, the mean ± SD daily insulin units were 71.1 ± 51.2, 63.1 ± 36.9, and 51.4 ± 26.6, respectively (p = 0.14). Among subjects with a baseline A1c ≥ 8.0%, the mean ± SD daily insulin units at follow-up also differed significantly (p = 0.04): non-Hispanic whites = 80.3 ± 51.3, Hispanics = 63.0 ± 34.0, and African Americans = 47.8 ± 23.1.

We found that race/ethnicity, BMI, baseline A1c, and use of any other oral hypoglycemic medication were significant predictors of daily insulin units on multivariate linear regression analyses (Table 4). African-Americans received an insulin dose that was an average of 17.8 units less (P = 0.01) and Hispanics an average of 10.5 units less (P = 0.04) than non-Hispanic whites. No significant interactions were observed. When the adjusted model was limited to those with baseline A1c ≥ 8%, African-Americans received an insulin dose that was an average of 26.5 units less (P = 0.01) and Hispanics an average of 15.9 units less (P = 0.03) than non-Hispanic whites. These differences persisted after adjusting for practice site.

## Discussion

We evaluated racial/ethnic differences in glycemic control, medical therapy, and control of cardiovascular disease risk factors in insulin-treated Southwest American veterans with type 2 diabetes. We found that African Americans and Hispanics had poorer glycemic control and received less intensive insulin treatment, particularly African-Americans with A1c ≥ 8.0% who received over 25 units of insulin less per day less than non-Hispanic whites. We found that blood lipids and blood pressure control were close to ADA target values for all racial/ethnic groups. This finding is consistent with previous findings that failure to intensify insulin treatment contributed to poor glycemic control in urban African-Americans [32]. This under-treatment is an important health problem, as evidence suggests that African Americans respond better to insulin treatment [33].

Several psychosocial factors and barriers to care or self-care varied among the racial/ethnic groups in this study, and could be related to the observed disparities in glycemic control. Hispanics were the most disadvantaged group in terms of language preference and education and scored higher on the depression inventory. These factors may explain their performance on the MMSE and Diabetes Knowledge tests – tasks that required comprehension of complex instructions. On the other hand, African-Americans had more favorable responses in several areas rated by the Diabetes Care Profile. We could not readily attribute poorer glycemic control to a negative outlook in this group.

Other investigators have found that racial/ethnic differences in attitudes towards type 2 diabetes might contribute to poor outcomes [34,35]. Possibly, instruments specifically developed for that purpose might have explained some of the effects of race/ethnicity on glycemic control and treatment intensity. For example, the Veterans Ecocultural Self Report [36] adaptation measure better explains the effect of minority status on glycemic control than does Hispanic ethnicity. Our inability to identify these factors may also be due to the fact that our instrument did not specifically target insulin therapy. Hunt and associates [37] used an open-ended interviewing technique to examine the attitudes of 44 low-income Mexican Americans towards insulin therapy. Negative aspects were much more frequently discussed than positive aspects and focused on anxiety about pain, proper techniques, disrupting daily activities, low blood sugars, and other complications of therapy. The subjects also expressed concern that previous treatment efforts had failed and that the disease had progressed into a more serious phase. Our study suggests that integrating an abbreviated interview technique could be considered for future studies of minority subjects with an inadequate response to insulin therapy.

Potential cultural barriers to a more intensive insulin regimen in minorities also include: 1) a greater aversion to parenteral injections or to multiple injections; 2) greater fear of hypoglycemic events; 3) greater aversion to glucose monitoring; and 4) cultural barriers relating to images of wellness, such as a cultural aversion to public display of illness.

This study has some potential limitations. We cannot explain why minorities with poor glycemic control did not receive higher insulin doses. One possibility is a selection bias that precluded the enrollment of minorities with more aggressive insulin regimens and better glycemic control. We randomly selected subjects from a sample frame generated from administrative pharmacy files; however, a number of eligible patients refused to participate. Because participation in this study required time, travel, disruption of daily routines, and some discomfort, even minor cultural barriers may have played a role in the loss of more intensively-treated minorities. We were unable to ascertain the race/ethnicity of non-participants. Since provider adjustments to insulin dose are not captured in the administrative pharmacy database, we relied on self-report from patients for current insulin units per day via structured interviews with research coordinators. While this was the best practical source of information, it is possible that minority patients systematically under-reported their insulin dose. Another possibility for the difference in treatment intensity is that minority patients had fewer clinic visits. We were unable to assess this because we did not have data on the number of clinic visits since being started on insulin. However, it is unlikely that an effect from fewer visits would persist over an average of 8 years of insulin treatment. Additionally, given the equal access to VA health care shared by all veterans, the frequency of clinic visits would not likely be an explanation for differential treatment, but rather a marker for confounding socioeconomic or psychological factors. Finally, because of the population studied, our results may be less generalizable to younger patients, non-veterans, or women.

Insulin treatment may have been less intensive in minorities for clinical reasons. Hypoglycemia is a major deterrent to tight glycemic control. Although age has been identified as a risk factor for drug-induced hypoglycemia [38], the roles of race and ethnicity have not been established. Possibly, some providers perceive a higher hypoglycemia risk for minority patients that may affect treatment intensity. This hypothesis requires further evaluation. The risk of hypoglycemia could be affected by dietary habits that are influenced by race and culture. Another possibility is racial/ethnic differences in the intensity of glucose monitoring. Because monitoring is used to titrate insulin doses, targets may not have been reached in those who tested less frequently. In 1993, Harris and co-workers [39] reported data from the 1989 National Health Interview Survey on 2,405 diabetic patients ≥ 18 years of age. They found that African-Americans were 60% less likely to test their blood glucose at least once daily compared to non-Hispanic whites and Hispanics. The effect of race/ethnicity was independent of age, insulin use, education, intensity of physician visits, or diabetes education. Unfortunately, we did not obtain information on monitoring practices or the rate of hypoglycemia when patients entered this study.

Finally, it should be noted that the differences among the racial/ethnic groups were limited to insulin use. We found no racial/ethnic differences in the use of oral hypoglycemic medications or in the control level of 5 other risk factors for cardiovascular disease (weight exercise, smoking status, lipids, and blood pressure). In contrast, Heisler and co-workers [40] found that African American veterans with type 2 diabetes were more likely to have poor lipid and blood pressure control compared to white veterans, although there was no difference in intensity of treatment for those in poor control (the intensity of the glycemic regimen was not measured). Our study may have been underpowered to detect small differences in lipids, blood pressure, and other risk factors. However, the finding of comparable control across racial/ethnic groups suggests that subjects did not face substantial access barriers and that providers were addressing these cardiovascular risk factors. However, glycemic control and insulin treatment require more motivation and patient education than other aspects of cardiovascular disease risk factor control. Failure to achieve treatment goals may have been due to specific problems with the patient-provider interaction. One possibility is that the patient was not able to establish an effective therapeutic relationship with a provider of another race. A second possibility is that the providers were not aware of the specific needs of minorities with diabetes [41]. Providers often address multiple acute and chronic conditions during medical encounters; this may present a barrier for consistently providing preventive services and optimal disease management for diabetes, particularly if minority patients have more comorbidity than non-Hispanic white patients [42,43]. Although provider effects are an important determinant of diabetes control, we did not have sufficient power to model this factor in our analyses.

## Conclusion

In summary, insulin-treated veterans who are minorities may have an increased risk of poor glycemic control and receiving lower doses of insulin. African-Americans in this sample were the most likely patients to experience this problem, while Hispanics had an intermediate risk. No racial or ethnic differences were found for the control of other cardiovascular disease risk factors. The disparities in glycemic control and treatment intensity could not be explained by the socioeconomic barriers, attitudes, level of knowledge, depression, cognitive dysfunction, or social support rated by the instruments in this study. The treatment disparity could be due to provider behaviors and/or patient behaviors or preferences. Further research with larger sample sizes and more geographically diverse populations are needed to confirm our findings and to elucidate the reasons for any observed disparities.

## Competing interests

The author(s) declare that they have no competing interests.

## Authors' contributions

CSW served as data manager and analyst and drafted the manuscript. JHS participated in study conception, design, management, and interpretation. WCD participated in study conception, design, management, and interpretation. RMH participated in study conception, design, and interpretation, and helped to refine the manuscript. MJM participated in study conception and design, and helped to refine the manuscript. GHM conceived of and designed the study, managed it as PI, lead the statistical analysis, and helped to draft the manuscript. All authors read and approved the final manuscript.

## Pre-publication history

The pre-publication history for this paper can be accessed here:



## Figures and Tables

**Table 1:** Sociodemographic and clinical features by race and ethnicity

	Non-Hispanic white (n = 226)	Hispanic (n = 72)	African-American (n = 35)	P-value
**Demographics**				
Age: mean ± SD years	65.5 ± 9.4	64.9 ± 10.1	63.3 ± 10.8	NS
Female	5.3%	1.4%	0%	NS
Married	56.6%	66.7%	62.9%	NS
**Disease history**				
Diabetes duration: mean ± SD years	14.1 ± 9.5	15.4 ± 10.5	15.5 ± 10.6	NS
Duration of medical treatment: mean ± SD years	12.3 ± 9.0	14.1 ± 10.5	12.6 ± 10.0	NS
Duration of insulin treatment: mean ± SD years	8.0 ± 7.7	7.8 ± 8.1	8.6 ± 8.2	NS
**Complications**				
Neuropathy	59.7%	63.9%	51.4%	NS
Peripheral vascular disease	35.8%	41.7%	28.6%	NS
Angina or chest pain	35.0%	23.6%	40.0%	NS
Myocardial infarction	33.6%	26.4%	17.1%	NS
Retinopathy	31.9%	21.4%	34.3%	NS
Stroke	13.7%	13.9%	11.4%	NS
Extremity amputation	6.6%	8.3%	14.3%	NS
Chronic renal disease	3.5%	0%	2.9%	NS
Blindness	1.3%	1.4%	0%	NS
Any microvascular complication	67.7%	69.4%	71.4%	NS
Any macrovascular complication	66.4%	61.1%	57.1%	NS
**Disabilities**				
Work	55.8%	55.6%	31.4%	0.03
Household projects	56.6%	47.2%	45.7%	NS
Yard work	40.3%	33.3%	25.7%	NS
Exercise	28.4%	19.4%	25.7%	NS
Cooking	11.1%	9.7%	5.7%	NS
Shopping	9.7%	12.5%	11.4%	NS
Self-care	4.0%	5.6%	2.9%	NS
Any of the above	72.1%	73.6%	54.3%	NS
**Another person:**				
Measures blood sugars	1.3%	0%	5.7%	NS
Administers insulin	4.0%	0%	5.7%	NS
**Family circumstances**				
Head of household	89.3%	97.2%	94.3%	NS
Size of household: mean ± SD	1.96 ± 1.16	2.35 ± 1.60	2.03 ± 0.92	0.04
Number of dependents: mean ± SD	0.57 ± 0.90	0.83 ± 0.90	0.69 ± 0.80	0.02
Family member with alcoholism	6.6%	2.8%	8.6%	NS
Family member with disability	50.9%	41.7%	45.7%	NS
Caregiver to another person	39.4%	30.6%	37.1%	NS
				
**Occupational history**				
Employed ≥ 8 hours/week	24.3%	25.0%	37.1%	NS
Work hours from 5:00 pm to 8:00 am	9.3%	9.7%	14.3%	NS
Variable job site	7.5%	9.7%	14.3%	NS
				
**Geographic barriers**				
Distance to hospital (miles): mean ± SD	20.3 ± 28.3	21.1 ± 30.3	11.3 ± 7.3	0.07*
Drives automobile	88.1%	93.1%	85.7%	NS
				
**Medical treatment**				
*Insulin *(mean ± SD)				
Units per day	70.6 ± 48.8	58.4 ± 32.6	53.1 ± 36.2	<0.01^†^
Number of injections per day	2.09 ± 0.66	2.11 ± 0.52	1.91 ± 0.45	NS
Number of preparations	1.43 ± 0.54	1.43 ± 0.50	1.29 ± 0.46	NS
*Oral medications*				
% subjects using	33.2%	34.7%	31.4%	NS
Number of dosing times	0.65 ± 1.00	0.72 ± 1.00	0.66 ± 1.00	NS
				
**BMI (kg/m**^2^**): **mean ± SD	32.3 ± 6.2	30.9 ± 5.7	31.1 ± 4.5	NS
				
**Exercise (met-hours per week): **mean ± SD	64.1 ± 63.9	74.3 ± 77.7	72.7± 48.8	NS
				
**Current smoker**	19.9%	25.0%	22.9%	NS
				
**Entry HbA1c (%): **mean ± SD	7.86 ± 1.41	8.16 ± 1.61	8.84 ± 2.87	0.05†
				
**Blood lipids: **mean ± SD				
Cholesterol (mg/dL)	189 ± 46	192 ± 57	196 ± 35	NS
Triglycerides (mg/dL)	229 ± 190	225 ± 159	164 ± 96	0.08†
HDL (mg/dL)	39.5 ± 11.6	40.0 ± 11.5	44.0 ± 13.4	NS
LDL (mg/dL)	109 ± 42	105 ± 38	121 ± 36	NS
				
**Blood pressure: **mean ± SD				
Systolic (mm Hg)	138 ± 18	138 ± 17	133 ± 20	NS
Diastolic (mm Hg)	74 ± 10	75 ± 10	77 ± 10	NS

Abbreviations:

SD = standard deviation

BMI = Body Mass Index

HbA1c = Glycosylated hemoglobin

*Kruskal-Wallis one-way analysis of variance by ranks

† Brown-Forsythe test

**Table 2:** Psychological features by race and ethnicity*.

	Non-Hispanic white (n = 226)	Hispanic (n = 72)	African-American (n = 35)	P-value
Preference for English	98.7%	83.3%	100%	<0.001
Education (years)	13.7 ± 2.8	11.9 ± 4.0	12.9 ± 2.0	<0.001^†^
Cognitive Deficit mean^§^	28.3 ± 2.0	26.4 ± 3.5	27.7 ± 1.8	<0.001
Family Behavior Checklist score^||^	6.16 ± 6.92	6.33 ± 6.88	8.26 ± 5.92	NS
Diabetes Knowledge score^¶^	67.4 ± 13.9	56.3 ± 17.0	62.1 ± 16.8	<0.001
Depression score**	7.60 ± 6.58	9.42 ± 7.14	6.03 ± 5.63	0.03^‡^
Diabetes Care Profile scores^††^				
Problems with glycemic control	3.98 ± 0.66	3.93 ± 0.56	4.27 ± 0.58	0.02^‡^
Social and personal impact	3.47 ± 0.82	3.39 ± 0.84	3.73 ± 0.72	NS
Positive attitudes	3.10 ± 0.72	2.99 ± 0.72	3.12 ± 0.94	NS
Negative attitudes	3.53 ± 0.80	3.24 ± 0.90	3.80 ± 0.83	0.008^‡^
Perceived ability to do self-care	3.00 ± 0.74	3.00 ± 0.87	3.53 ± 0.74	0.001^‡^
Importance of self-care	4.24 ± 0.58	4.29 ± 0.62	4.45 ± 0.66	NS
Adherence to self-care	3.49 ± 0.74	3.51 ± 0.77	3.80 ± 0.79	NS
Adherence to diet	2.65 ± 0.94	2.65 ± 0.96	3.08 ± 0.81	0.04^‡^
Barriers to taking medications	4.57 ± 0.45	4.55 ± 0.56	4.59 ± 0.52	NS
Barriers to exercise	4.04 ± 0.80	4.04 ± 0.80	4.37 ± 0.64	NS
Barriers to monitoring	4.63 ± 0.46	4.50 ± 0.60	4.38 ± 0.83	NS
Understanding of objectives	3.44 ± 0.81	3.22 ± 0.81	3.57 ± 0.83	NS
Perception of long-term benefits	4.38 ± 0.68	4.29 ± 0.86	4.15 ± 0.96	NS
Social support	3.82 ± 0.60	3.87 ± 0.63	3.94 ± 0.63	NS

* Values are mean ± SD unless otherwise indicated

^†^Brown-Forsythe test

^‡^Kruskal-Wallis one-way analysis of variance by ranks

^§^Mini-Mental State Examination: 30-point scale, higher score more favorable

^||^Scale ranging from -44 to 38, higher score more favorable

^¶ ^University of Michigan Diabetes Knowledge Test: percentage correct, higher score more favorable

**Geriatric Depression Scale: 30-point scale, lower score more favorable

^†† ^Scale ranging from 1 to 5, higher score more favorable

**Table 3:** Daily insulin units by baseline hemoglobin A1c and race/ethnicity

A1c category	Non-Hispanic white		Hispanic		African-American		All subjects	
	
	N	Mean ± SD daily units	N	Mean ± SD daily units	N	Mean ± SD daily units	N	Mean ± SD daily units
< 7.0%	66	62 ± 52	14	54 ± 39	11	56 ± 50	91	60 ± 49
7.0%–7.9%	69	68 ± 46	22	63 ± 34	6	50 ± 22	98	66 ± 42
≥ 8.0%	91	79 ± 48	35	57 ± 29	18	53 ± 31	144	70 ± 44

**Table 4:** Regression model predicting daily insulin units.

Predictor	Coefficient	95% Confidence Interval	P value
Hispanic*	-10.5	-20.6, -0.46	0.04
African American*	-17.8	-31.4, -4.3	0.01
BMI^† ^(kg/m2)	3.4	2.7, 4.2	< 0.001
Baseline A1c^‡ ^(%)	3.0	0.50, 5.5	0.02
Age (years)	-0.36	-0.81, .094	0.12
Metformin	-9.1	-20.1, 1.9	0.10
Other OHM^§^	-32.0	-42.4, -21.5	< 0.001
Constant	-30.6	-80.7, 19.5	0.23

Number of observations = 327, R^2 ^= 0.336 (P < 0.0001)

* Effect in reference to non-Hispanic whites

† Body Mass Index

‡ Hemoglobin A1C

§Oral hypoglycemic medication
